# The Association of Weight Categories in Adolescence with Cardiovascular Morbidity in Young Adult Israeli Arabs—A Nationwide Study

**DOI:** 10.3390/jcm13185382

**Published:** 2024-09-11

**Authors:** Yulia Treister-Goltzman, Dan Nemet, Idan Menashe

**Affiliations:** 1 Department of Family Medicine and Siaal Research Center for Family Practice and Primary Care, The Haim Doron Division of Community Health, Faculty of Health Sciences, Ben-Gurion University of the Negev, P.O. Box 653, Beer-Sheva 84105, Israel; 2 Clalit Health Services, P.O. Box 16250, Tel Aviv 62098, Israel; 3 Child Health and Sports Center, Meir Medical Center, Kfar-Saba 4428164, Israel; dan.nemet@clalit.org.il; 4 School of Medicine, Tel Aviv University, P.O. Box 39040, Tel Aviv 6139001, Israel; 5 Department of Public Health, Faculty of Health Sciences, Ben-Gurion University of the Negev, Beer-Sheva 84105, Israel; idanmen@bgu.ac.il

**Keywords:** childhood obesity, diabetes mellitus type 2, hypertension, ischemic stroke, heart failure, cardiovascular risk factors

## Abstract

**Objectives.** The health consequences of adolescent obesity are understudied in young adult Israeli Arabs. We aimed to evaluate the association of weight categories during adolescence with hypertension (HTN), diabetes mellitus type 2 (DM2), and the composite endpoint of ischemic stroke (IS), myocardial infarction (MI), and heart failure (HF) in young adult Israeli Arabs on a nationwide level. **Methods.** A retrospective cohort study of 53,726 Arab adolescents born from 1988–1992 was conducted. The cohort was followed, beginning with BMI measurements at ages 17–19 years, until whichever came first among the diagnosis of outcome disease, death, discontinuation of health insurance, or age of 30 years. **Results.** The incidence (95% CI) of HTN, DM2, and the composite endpoint of IS, MI, and HF was 138.2 (129.1–147.9), 136.7 (127.6–146.3), and 27.3 (23.3–31.7) cases per 10^5^ person-years, respectively. The risk for DM and HTN increased gradually, starting from the ‘overweight’ category, and reaching fully adjusted HRs (95% CI) of 2.80 (1.82–4.30), and 1.97 (1.31–2.96), respectively, in the ‘class 3 obesity’ category. The Hazard ratio (HR) for the composite endpoint, its incidence and components, was highest in the ‘overweight’ category (aHR of 1.64 (1.08–2.50)). **Conclusions.** The findings emphasize the long-term health consequences of adolescent obesity in early adulthood and, hence, the need for interventions aimed at reducing the rate of adolescent overweight and obesity. The finding of a very high rate of DM2 incidence in early adulthood, even among adolescents without obesity, necessitates an integrated public health approach to all risk factors to prevent DM2 in this population.

## 1. Introduction

The health consequences of adolescent obesity have been researched extensively in the Jewish population in Israel, based mainly on height and weight measurements from the medical examination prior to army recruitment, from which the Arab population is exempted. Among the multiple health ramifications of adolescent obesity, cardiovascular morbidity plays a lead role in terms of prevalence and strength of association. Adolescent obesity was found to be associated with cardiovascular comorbidity already in adolescence with an odds ratio (OR) of 2.1–3.4 for HTN and 15.1–38.0 for DM2, depending on obesity severity [1]. The prospective risk of adolescent obesity has been demonstrated in young adults. The Hazard ratios (HRs) for DM2 in early adulthood, with a mean age of diagnosis at 27 years, were 13.4–25.8 for men and 21.1–44.7 for women within mild to severe adolescent obesity groups, respectively [2]. The HR for the first ischemic stroke (IS) for individuals with obesity during adolescence was 3.4, with an increased risk seen even in the high normal and overweight groups [3]. Moreover, adolescent obesity among Jewish men was associated with cardiovascular disease-specific and all-cause mortality [4,5,6].

Variations in the prevalence rates for cardiovascular diseases in different ethnic groups have been described globally and in Israel [7,8]. These differences are explained by different risk factor profiles, among them genetic factors, smoking, HTN, dyslipidemia and DM2 [9,10]. Many of these risk factors are a direct consequence of obesity. Cultural norms, values, and practices also contribute to the risk of cardiovascular disease [10,11]. Therefore, the obesity-related population attributable risk for the development of these diseases varies among ethnicities [12]. In a pilot study on obesity prevalence and incident DM2 in young adult Israeli Negev Bedouins, the incidence rates for DM2 were even higher than those reported recently for Ethiopian immigrants in Israel, an ethnic group that is known for its high DM2 rates, especially among young and second-generation members of the community [13].

The Arab population of Israel has a lower life expectancy than the Jewish population. Several studies have demonstrated that disease-specific mortality from diabetes and heart and cerebrovascular diseases is the main reason for the increased mortality in this population [8]. Even after adjusting for education level, the gaps remain [8], highlighting the impact of additional factors on these discrepancies. Examining the contribution of adolescent obesity to the development of cardiovascular morbidity, especially at a young age, can help to understand its causes better and to guide preventive health initiatives in a more efficient way.

The goal of the present study was to examine the incidence of HTN, DM2, and the composite endpoints of IS, MI, and HF in young adulthood, and to evaluate the association of weight categories during adolescence with these diseases in young Israeli Arab adults on a nationwide level.

## 2. Methods

### 2.1. Study Design and Setting

This was a retrospective cohort study, based on the centralized computerized database of the “Clalit Health Services” (CHS), the major health sick fund which insures 52% of the Israeli population. The study population consisted of Arab adolescents born in 1988–1992 who had recorded measurements of weight and height at ages 17–19 years. We excluded participants with preexisting diseases relevant to each outcome and participants with major chromosomal anomalies and intellectual disabilities. The study period was from 1 January 2007 to 31 December 2022. It was divided into the exposure period from 1 January 2007 to 31 December 2011 (ages 17–19) and the follow-up period from 1 January 2007 to 31 December 2022. It continued from the date of the defining BMI measurement until whichever came first among the diagnosis of an outcome disease, death, discontinuation of insurance in the CHS, or age of 30 years. “Young adulthood” was defined for the purpose of this study as ages between 17–19 and 30 years for two reasons. The main reason was a technical constraint: the availability of the follow-up data after the defining BMI measurement in the computerized database ended at age 30 years for all participants. The second reason was the physiological and developmental plausibility of this definition, as it corresponds to the emerging early adulthood stage of the life course.

The data collected included socio-demographic data (age, sex, ethnic sector, district of residence, and socio-economic status, as defined in the CHS computerized database by zip code), height (cm), weight (kg), BMI, and BMI percentile of the adolescents, the recorded diagnoses of interest (HTN, DM2, IS, MI, and HF), and major chromosomal anomalies and intellectual disabilities. Additionally, the total number of insured Arab adolescents aged 17–19 years during the study period was obtained to assess the rate of missing data.

The study was conducted according to the guidelines of the Declaration of Helsinki. The Ethics Committee of Clalit Health Services approved the study (approval #0102-22-COM1, date: 11 December 2022) and exempted it from the requirement to obtain informed consent.

### 2.2. Definitions of Variables

BMI was defined as weight in kilograms divided by height squared in meters. Adolescent weight categories were defined as the percentiles determined by the U.S. Center for Disease Control and Prevention (CDC), which were validated for Israeli adolescents, as ‘underweight’ (BMI < 5th percentile), ‘normal weight’ (5th–84.9th percentile), ‘overweight’ (85th–94.9th percentile), ‘obesity’ (≥95th percentile, but not including ‘class 2’ and ‘class 3 obesity’). ‘Class 2 obesity’ was diagnosed if BMI reached ≥ 120% to <140% of the 95th percentile or BMI ≥ 35 to <40 kg/m^2^. ‘Class 3 obesity’ was diagnosed if BMI was ≥140% of the 95th percentile or BMI was ≥40 kg/m^2^.

The diagnosis of primary HTN (after exclusion of secondary HTN) was established by a combination of one of the ICD-9 codes of 401.0, 401.1, and 401.9 and the absence of the ICD-9 codes of 402, 403, 404, and 405, with an area under the curve of 90.1% for the diagnosis of HTN [14]. DM2 was diagnosed by an ICD-9 code of 250.X0 or 250.X2 and the absence of an ICD-9 code of 250.X1 or 250.X3. This combination had a diagnostic accuracy of 99% and an area under the curve of 99.8% for the diagnosis of DM2 [15]. We chose to examine IS incidence only, since at ages 30 and below the most common type of hemorrhagic stroke is structural vasculopathy/traumatic, which has no theoretical connection with obesity/cardiovascular risk factors [16]. The diagnosis of IS was made in the presence of one of the ICD-9 codes of 433.X1, 434.X1, and 436, which demonstrated a positive predictive value (PPV) of 70–91% and a negative predictive value (NPV) of 99–100% for the diagnosis of different types of IS (precerebral/cerebral occlusion and cerebrovascular disease) [17]. MI was diagnosed by an ICD-9 code of 410.X, with a previously demonstrated PPV of 95% and an NPV of 97% [18], and HF by the diagnostic codes of 428.X with a PPV 94% and an NPV of 93% (95% CI, 85–98%) [18]. The composite endpoint of IS, MI, and HF was determined if the participant developed at least one of these conditions, and their follow-up ended on the date of the earliest condition. Major chromosomal and other congenital anomalies were defined by ICD-9 codes of 758.0, 758.1, 758.2, 758.3, 758.5, 758.6, 758.7, 758.8, 758.9, 759.5, 759.6, 759.7, 759.8, and 759.9, and moderate to severe intellectual disabilities by ICD codes of 318 and 319. 

### 2.3. Statistical Analyses

Data cleaning was performed, and outlying BMI values were deleted. We compared the basic socio-demographic characteristics of adolescents with and without BMI measurements to assess possible selection bias. The study sample was finalized after the exclusion of patients with major chromosomal abnormalities and intellectual disabilities. For adolescents with more than one measurement of weight and height at ages 17–19, we chose the earliest BMI measurement for analyses. We characterized the baseline features of the study population using descriptive statistics. Kaplan–Meier survival curves were computed for adolescents in different weight categories for HTN and DM2. The Log-rank test was used to compare survival curves between the weight groups. A log minus log plot (LML plot) and a Schoenfeld residual test, which tests the difference between the observed explanatory variables and the estimates from a Cox model, were applied to validate the proportionality assumption. The curves of the LML plots did not intersect, and the results of the Schoenfeld residual test were insignificant for individual predictors and globally in all models (Table S1).

After making sure that the proportionality assumption held, Cox proportional Hazard models were used to estimate the HR and 95% CIs for incidental HTN and DM2, with the “normal” weight category serving as the reference group. Multivariable Cox regression models were adjusted for sex, socio-economic status, district of residency, and adult BMI. 

IS, MI, and HF are rare ramifications of obesity at ages younger than 30, so a composite endpoint of the three conditions was assessed in this study by the time-to-first-event method. The main justification for the combination of these events was a gain in statistical efficacy due to the rare individual events rate and the short length of the available follow-up. These three diseases, although distinct, share the most key pathophysiological mechanisms in the context of obesity, in particular abnormalities in adipokines (e.g., adiponectin and leptin), aberrant fatty acid uptake phenotype, and hyperinsulinemia, leading to both impaired myocardial metabolism and HF, and atherosclerotic plaque formation in IS and MI [19]. Even the genetic and molecular factors underlying these cardiovascular complications in obesity are inter-related [19]. Thus, combining these diseases when examining the cardiovascular consequences of obesity seemed plausible to us from a pathophysiological perspective. The time-to-first-event method has been used commonly for the analysis of composite endpoints; however, it has the inherent limitation of treating all contributory endpoints as having equal severity and only gives weight to the first endpoint encountered in time [20]. Thus, events that occurred earlier have more impact than events that occurred later [20]. Some participants in our study experienced several events that affected the composite endpoint (IS, MI, and HF) and incorporation of all events is meaningful. Therefore, in addition to the Cox proportional Hazard model for the composite outcome, we presented incidence rates for each of the individual components, and built a negative binomial regression which captures all events and hence tends to have more statistical power than time-to-first-event analysis [20]. This regression is considered better suited than Poisson for the events in clinical settings, in which variance of events is usually greater than the mean [19]. For the composite IS, MI, and HF outcome an additional adjustment was made for HTN and DM2. The population attributable risk percent (PAR%) for each outcome was calculated for excessive weight (overweight and all obesity categories together) as follows:

$$PAR\%=\frac{Pe(HR-1)}{Pe\left(HR-1\right)+1}*100$$

in which HR is the unadjusted HR for the outcome and Pe is the prevalence of the excessive weight at the beginning of the follow-up. Statistical significance was set at *p* < 0.05.

## 3. Results

Figure S1 presents a flowchart of the study cohort selection process. Of 126,778 Arab adolescents insured in CHS who reached ages 17–19 in 2007–2011, 55,050 had weight and height measurements in their medical records. After excluding individuals with outlying BMI values, major chromosomal anomalies, and moderate to severe intellectual disabilities, 53,726 adolescents comprised the final cohort. Table S2 shows that there were no major differences in socio-demographic characteristics between those with BMI measurements and those without them. Table S3 demonstrates the socio-demographic characteristics of the study participants. The mean age (SD) was 18.2 (0.9). Males constituted slightly less than half of the participants (47.3%), with higher percentages in the ‘underweight’ and all three ‘obesity’ categories. The mean (SD) of BMI and BMI percentile was 23.2 (4.4), and 50.1 (30.4), respectively, increasing through weight categories as expected. More than 60% of the adolescents belonged to the low socio-economic level, and less than one percent to the high. In all weight categories, most of the participants resided in Haifa and the Northern and Jerusalem districts (around 20% in each), except for the ‘underweight’ category, in which 20% of the participants resided in the Southern district. 

### 3.1. Incidence of HTN and DM2

Table S4 presents risk estimates of the associations between adolescent weight categories and HTN and DM2 incidence. After exclusion of patients with pre-existing morbidity, 53,525 and 53,489 adolescents constituted the cohorts for HTN and DM2, respectively.

**Figure 1:**
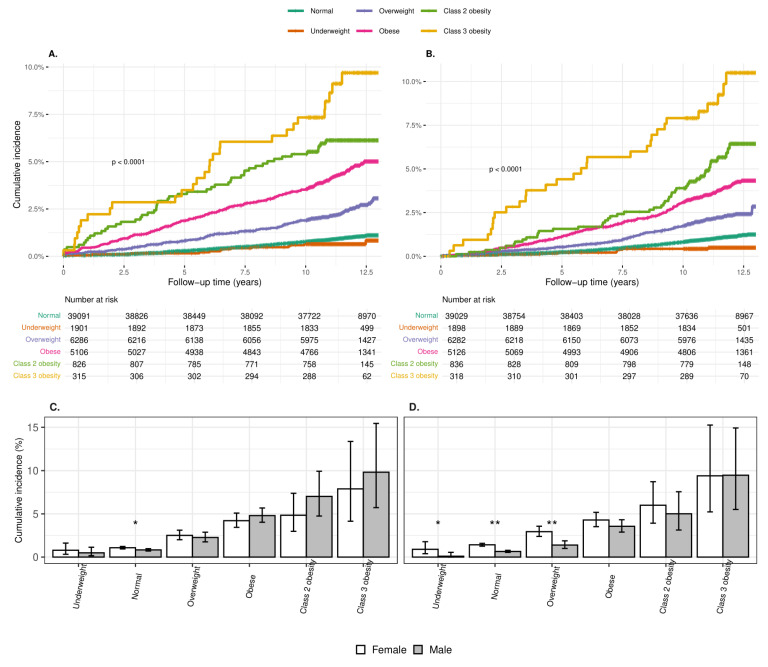
Incidence of hypertension and diabetes mellitus type 2 by weight group. Kaplan–Meier survival curves of incident hypertension (**A**) and diabetes mellitus type 2 (**B**) by weight groups. Cumulative incidence of hypertension (**C**) and diabetes mellitus type 2 (**D**) by weight group and sex. Underweight—BMI < 5th percentile, normal weight—BMI 5th–84.9th percentile, overweight—BMI 85th–94.9th percentile, obese—BMI ≥ 95th percentile, not including class 2 and class 3 obesity, class 2 obesity—BMI ≥ 120% to < 140% of the 95th percentile or BMI ≥35 to <40 kg/m^2^, class 3 obesity—BMI ≥ 140% of the 95th percentile or BMI ≥ 40 kg/m^2^. The cumulative incidence of hypertension was significantly higher among females in the ‘normal’ weight category * (*p* = 0.013), and of diabetes mellitus type 2 among females in ‘underweight’ * (*p* = 0.027), ‘normal’ ** (*p* < 0.001), and ‘overweight’ ** (*p* < 0.001) categories.

There were 848 incident cases of HTN in 613,409 person-years of follow-up. The mean follow-up for HTN varied from 11.0 to 11.6 years, with a shorter follow-up time for individuals in higher weight categories (*p* < 0.001). The mean age (SD) at diagnosis was 26.0 (4.3) years, but it was lower in the excessive weight categories. Adult BMI increased with increasing adolescent weight category. The incidence of HTN showed a graded increase by BMI groups from “underweight” to “class 3 obesity” (from 58.7 to 807.1 diagnoses per 10^5^ person-years, respectively). The results of the Kaplan–Meier survival analysis (Figure 1A) and Cox modeling (Table S4) further illustrated this trend. The crude HR (95% CI) for HTN gradually increased to 9.71 (6.61–14.26) in the “class 3” obesity category, compared to the reference “normal” weight category. When adjusted for gender, socio-economic status, district of residency, and adult BMI, a more moderate increase in HR (95% CI) was observed through weight categories, reaching 2.80 (1.82–4.30) in the “class 3” obesity category.

Eight hundred thirty-nine participants developed incident DM2 during 613,790.8 person-years of follow-up. The mean (SD) follow-up was shorter in the “class 2” and “class 3” obesity categories 11.5 (1.6). The mean age (SD) at diagnosis was 25.5 (3.3) years, with no significant differences between the weight groups. The incidence of DM2 increased consistently from 40.7 to 854.2 per 10^5^ person-years between the “underweight” and “class 3” obesity categories. The Kaplan–Meier survival analysis (Figure 1B) demonstrated a separation of the curves of the weight groups in the first years of follow-up. The crude and aHR (95% CI) for DM2 increased gradually, reaching 9.32 (6.43–13.50) and 1.97 (1.31–2.96), respectively, in the “class 3” obesity category (Table S4). 

The cumulative incidence (%) (95% CI) of HTN to age 30 years was similar in females and males for the total population (1.60 (1.45–1.75) and 1.57 (1.42–1.73), respectively, *p* = 0.830) and in most of the weight categories, other than the “normal” category, where it was higher in females than in males at 1.08 (0.94–1.23) vs. 0.83 (0.70–0.97), respectively, (*p* = 0.013) (Figure 1C).

The cumulative incidence (%) (95% CI) of DM2 to age 30 was significantly higher in females than in males for the total population (1.94 (1.78–2.10) vs. 1.16 (1.03–1.30), respectively, *p* < 0.001) and in the “underweight,” “normal,” and “overweight” categories, but in all “obesity” categories the difference was not significant (Figure 1D).

### 3.2. Composite Outcome of IS, MI, and HF

**Figure 2:**
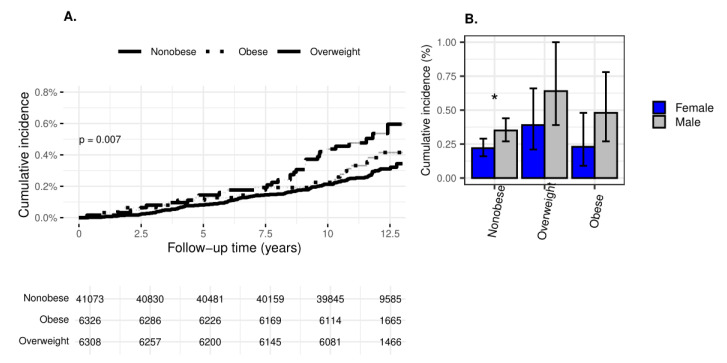
Incidence of the composite endpoint of ischemic stroke, hypertension, and diabetes mellitus type 2 by weight group. (**A**) Kaplan–Meier survival curves of the incident composite endpoint of ischemic stroke, myocardial infarction, and heart failure by weight group. (**B**) Cumulative incidence of the incident composite endpoint of ischemic stroke, myocardial infarction and heart failure by weight group and sex. Nonobese—BMI < 85th percentile, overweight—BMI 85th–94.9th percentile, obese—BMI ≥ 95th percentile. * The cumulative incidence of the composite endpoint was significantly higher in males than in females only in the ‘nonobese’ category (*p* = 0.019).

Of 53,707 participants without prior IS, MI, and HF, 169 participants developed at least one of the conditions during 619,325.5 person-years of follow-up (Table S5). The incidence was highest in the “overweight” category at 44.3 per 10^5^ person-years. The mean (SD) follow-up was 11.5 (1.5), higher in the “obese” category at 11.6 (1.6(, *p* < 0.001, and the mean age at diagnosis was 25.4 (3.2), with no statistically significant difference between the weight groups. Compared to the reference “nonobese” category, there was a statistically significant higher risk for the composite endpoint in the “overweight” category, with unadjusted and adjusted for socio-demographic characteristics and BMI HRs (95% CI) of 1.85 (1.25–2.73(, *p* = 0.002, and 1.70 (1.12–2.57(, *p* = 0.012, respectively. When further adjusted for HTN and DM2, the adjusted Hazard ratio (aHR) (95% CI) in this category remained similar at 1.64 (1.08–2.50, *p* = 0.020). Interestingly, there was no increased risk for the composite morbidity endpoint in the “obese” adolescent weight category compared to the reference “nonobese” category. The Kaplan–Meier survival plot (Figure 2A) depicted a clear separation of survival curves, higher for the “overweight” category, between 5.0–7.5 years of follow-up, i.e., at a young age of the participants.

The negative binomial regression, which incorporated the number of events as a dependent variable, showed that compared to the reference “nonobese” category, in the “overweight” category the RR for the number of cardiovascular events increased (95% CI) with 1.79 (1.17–2.67, *p* = 0.006) in the unadjusted model, 1.68 (1.07–2.58, *p* = 0.020) in the model adjusted for socio-economic variables and adult BMI, and 1.64 (1.05–2.51, *p* = 0.025) in the model adjusted for HTN and DM2 (Table S5). The number of additional cardiovascular events did not increase significantly in the “obese” category.

In the analyses of risk estimates for each component (Table S5), the highest incidence (95% CI) of 12.3 (9.7–15.3 per 10^5^ person-years) and the youngest mean (SD) age at presentation of 25.3 (3.1) were seen for HF, and the lowest incidence (95% CI) of 5.6 (3.9–7.9) per 10^5^ person-years was seen for MI. Among different weight categories, the highest incidence (95% CI) for all three outcomes was in the “overweight” category, at 22.1 (12.6–35.9), 8.3 (3.0–18.0), and 19.3 (10.6–32.5) per 10^5^ person-years for IS, MI, and HF, respectively, indicating that the finding of a higher risk in the “overweight” category was true for the individual components as well.

The cumulative incidence of the composite outcome (95% CI) was higher among males than females in the total population at 0.40 (0.32–0.48) vs. 0.24 (0.19–0.30), *p* = 0.001 and in the “nonobese” weight category (Figure 2B).

### 3.3. Population Attributable Risk

The fractions of incident cardiovascular outcomes that were attributed to excess weight were 54.3% for HTN, 42.9% for DM2, and 12.3% for the composite endpoint of IS, MI, and HF, based on the observed prevalence of 23.5% of BMI < 85th percentile during the exposure period.

## 4. Discussion

In this nationwide population-based cohort study we found a significant association between adolescent weight categories and incident cardiovascular risk factors (HTN and DM2) and even target organ damage (IS, MI, and HF) at age 30 and below in the Israeli Arab population. The association of obesity with HTN and DM2 is apparent during adolescence and is well recognized [1,21,22]. The cohorts in our study consisted of adolescents without a pre-existing comorbidity of HTN and DM2, and the Kaplan–Meier survival analysis demonstrated a constant escalation in risk during young adulthood, proportional to the increase in weight category. One of the goals of our study was to examine the strength of this relationship, due to the reports of its variation in different ethnicities [23,24]. There is a noticeable difference between unadjusted and adjusted for adult BMI and for socio-demographic characteristics HRs for both HTN and DM2. The weight group trajectory from adolescence is an important determinant of the risk for the development of HTN and DM2 in adulthood [25] and explains much of the differences between unadjusted and adjusted HRs. Both unadjusted and adjusted HRs for HTN and DM2 in our study population were comparable to that reported in the world for the overweight category, but much lower than reported in other populations, including Jews in Israel, for the severe obesity categories [2,25,26,27]. It is noteworthy that the overall incidence of DM2 among young Arab adults in our study is one of the highest reported in the world [28], and much higher than that reported for the Jewish population of the same age [2]. The high incidence of DM2 even in adolescents with normal BMI, which served as a reference group, explains the relatively low HRs for DM2 in the obesity groups. The incidence of HTN was lower than reported in some trials, but higher than reported in others [26,29]. Previous reports from Israel documented higher prevalence rates for both chronic conditions in the Israeli Arab population compared to the Israeli Jewish population [30,31]. 

The incidence of the composite IS, MI, and HF and its individual components was highest in the “overweight” category. This was the only category where the HR for the composite endpoint was significantly higher than in the reference “nonobese” category. The “obese” category did not entail an increased risk for this composite outcome. Most studies that assessed these cardiovascular outcomes had longer follow-up periods and demonstrated a rise in the rate of diagnoses after 20 years of follow-up, surging at ages 40–50 [3,32,33,34,35,36]. All these studies found a constant incremental increase in cardiovascular outcomes with increase in weight category. The shorter follow-up period in our study can explain the discrepant findings. Another possible explanation for the increased risk in the “overweight” category alone is that it is unique to the specific ethnic group in our study. Some researchers claim that adult weight groups have a much greater effect on cardiovascular morbidity than adolescent weight groups [37]. In our study, after adjustment for adult BMI, the “overweight” category in adolescence remained a significant independent risk factor for the composite endpoint. Furthermore, adjustment for HTN and DM2, which in some opinions are the main mediators of the association of excess weight and cardiovascular outcomes [38], hardly affected the strength of the association, providing further evidence for the independent association of the “overweight” category and end-organ damage. Although HF is usually considered a consequence of major risk factors, such as HTN, DM2, often through the development of ischemic heart disease [39], a growing body of evidence shows an independent effect of obesity, including adolescent obesity on its development [36,40,41]. Indeed, probably due to its multiple etiology, HF had the highest incidence among the individual components of the composite endpoint in our study.

The unique cultural health characteristics of this population could affect the results. Less than one third of Arab Israelis take part in regular physical activity and an increasing number consume processed foods due to the influence of Western diets [31]. These facts explain, at least in part, the overall high incidence of DM2 in general and even among young Arabs with a normal BMI in our study, resulting in relatively low HRs for DM2 in the obesity groups. The same factors could affect the rates of IS, MI, and HF in the “nonobese” and in the ”overweight’” and “obese” weight categories.

### Limitations and Strengths

The first limitation of our study is basing the definition of exposure on BMI from medical records, which may have led to selection bias. On the other hand, considering that the recommendation to measure height and weight for BMI for all adolescents aged 14–19 years has been an integral part of the Israel National program for quality indicators in the community since 2007, the missing BMI measurements could be considered as ‘missing at random.’ Furthermore, we demonstrated similarity in the socio-demographic characteristics between the adolescents with and without measurements in our study. The second limitation is that the data on the investigated comorbidities were based on the recorded diagnoses. Medical surveillance could have yielded more accurate diagnoses, although this approach was impossible in a study based on a large nationwide database. The specific ethnic nature of the study population limits its generalizability. On the other hand, the goal of our study was to assess the impact of weight categories in this specific ethnic population. While the study findings could not be extrapolated to the general population, they are very relevant locally due to the study’s national coverage. Moreover, they might be relevant to some populations in other countries with similar genetic and cultural characteristics (i.e., the Arab populations in Western countries).

The main strength of this study is that it was based on a nationwide, representative, large, and reliable database, with a large number of observations. Another strength is that the weight and height measurements and diagnosis recordings were done by health care professionals, not self-reported. The allocation to ‘class 2’ and ‘class 3’ obesity categories made it possible to identify associations of HTN and DM2 specific to these extreme degrees of obesity. A historically prospective design of the study, where the data on exposure were documented before the outcome, enabled us to determine the direction of the association, which is not possible in a cross-sectional study design. 

## 5. Conclusions

Our study demonstrated a high incidence of cardiovascular risk factors and target organ damage in the Israeli Arab population that is seen already in early adulthood (ages 30 and less) and their clear association with excess weight. For HTN and DM2, the risk increased gradually through weight categories, peaking in ”class 3 obesity”, while for the composite endpoint of IS, MI, and HF, the ”overweight” category represented the only risk-group. These findings, along with the finding that a significant fraction of the incident cases of morbidity could be attributed to excess weight, highlight the need for interventions aimed at reducing the rate of adolescent overweight and obesity. The finding of a very high rate of DM2 incidence in early adulthood, even among adolescents without obesity, necessitates public health and integrated approaches to all risk factors to prevent DM2 in this population.

## Data Availability

The data that support the findings of this study are available from “Clalit Health Services”. Restrictions apply to the availability of these data, which were used under license for this study. The data are available only with the permission of the “Clalit Health Services”.

## Supplementary Materials

The following supporting information can be downloaded at: https://www.mdpi.com/article/10.3390/jcm13185382/s1, Table S1: Results of the Schoenfeld residual test for proportionality assumption testing. Table S2: Comparison of adolescents with and without BMI measurements;; Table S3: Baseline characteristics of the study cohort; Table S4: Risk estimates of the association between adolescent weight category and incident hypertension and diabetes mellitus type 2 in young adulthood; Table S5: Risk estimates of the association between adolescent weight category and incident ischemic stroke, myocardial infarction, and heart failure in young adulthood. Figure S1: Flowchart of the study cohort selection.

## Abbreviations

HTN	Hypertension
DM2	Diabetes mellitus type 2
IS	Ischemic stroke
MI	Myocardial infarction
HF	Heart failure
HR	Hazard ratio
RR	Relative risk
OR	Odds ratio
CI	Confidence interval
BMI	Body mass index
CHS	Clalit Health Services
CDC	The U.S. Center for Disease Control and Prevention
ICD	International Classification of Diseases
PPV	Positive predictive value
NPV	Negative predictive value
PAR%	Population attributable risk percent
