# Supplementary material for: The Association of Weight Categories in Adolescence with Cardiovascular Morbidity in Young Adult Israeli Arabs—A Nationwide Study
Source: J Clin Med. 2024 Sep 11;13(18):5382. doi: 10.3390/jcm13185382 (PMC11432042; doi:10.3390/jcm13185382)

Table S1. Results of the Schoenfeld residual test for proportionality assumption testing

**Predictor**	**χ2**	**Degrees of freedom**	**p-value**
Model for Diabetes Mellitus			
Weight categories	7.35	5	0.196
Sex	1.47	1	0.226
Socio-economic status	0.92	3	0.822
Adult BMI	2.81	1	0.094
District of residency	3.17	6	0.787
Global	15.72	16	0.473
Model for Hypertension			
Weight categories	8.04	5	0.154
Sex	2.36	1	0.124
Socio-economic status	0.58	3	0.901
Adult BMI	0.00	1	0.995
District of residency	7.34	6	0.291
Global	18.32	16	0.305
Model for the composite endpoint			
Weight categories	0.03	2	0.986
Sex	1.84	1	0.175
Socio-economic status	2.95	3	0.399
Hypertension	3.65	1	0.056
Diabetes Mellitus	0.01	1	0.908
Adult BMI	1.84	1	0.175
District of residency	9.15	6	0.165
Global	19.47	15	0.193

Table S2. Comparison of adolescents with and without BMI measurements

Variable	Adolescents, who have measurements (N=54,627)	Adolescents who have no measurements (N=72,151)
**Sex (male),**		
N (%)	25,830 (47.3)	37,678 (52.2)
**Socio-economic status,** N		
(%):	34,009 (62.3)	42,590 (59.0)
low	13,910 (25.5)	20,522 (28.4)
medium	341 (0.6)	667 (0.9)
high	6,367 (11.7)	8,372 (11.6)
no data		
**District of residency,** N		
(%):	11,403 (20.9)	16,052 (22.2)
Northern	13,241 (24.2)	17,712 (24.5)
Haifa	9,287 (17.0)	10,415 (14.4)
Sharon-Shomron	935 (1.7)	1,144 (1.6)
Central	1,751 (3.2)	1,395 (1.9)
Dan-Petah-Tikva	9,626 (17.6)	15,371 (21.3)
Jerusalem	8,384 (15.3)	10,062 (13.9)
Southern		

Table S3. Baseline characteristics of the study cohort.

	Weight category							
	Underweight (N=1,909)	Normal (N=39,176)	Overweight (N=6,311)	Obese (N=5,164)	Class 2 Obesity (N=841)	Class 3 Obesity (N=325)	P	Total population (N=53,726)
Age of BMI measurement, years:								
Mean (SD)	18.1 (0.8)	18.3 (0.9)	18.3 (0.9)	18.1 (0.8)	18.3 (0.9)	18.4 (0.9)	<0.001[a]	18.2 (0.9)
Median (IQR)	18.0 (17.4-18.6)	18.1 (17.4-18.9)	18.1 (17.4-18.9)	17.9 (17.3-18.5)	18.2 (17.5-19.0)	18.4 (17.6-19.2)		18.1 (17.5-18.8)
Sex (male)								
N (%)	1,021 (53.5)	18,086 (46.2)	2,961 (46.9)	2,743 (53.1)	420 (49.9)	172 (52.9)	<0.001[b]	25,403 (47.3)
BMI, kg/m^2								
Mean (SD)	16.9 (1.0)	21.5 (2.0)	26.6 (1.3)	30.4 (2.0)	36.9 (1.3)	44.6 (4.6)	<0.001[a]	23.2 (4.4)
Median (IQR)	16.9 (16.3-17.5)	21.5 (20.0-23.1)	26.4 (25.6-27.3)	30.1 (28.6-31.7)	36.6 (35.5-37.7)	43.0 (40.6-45.4)		22.3 (19.9-24.7)
BMI percentile								
Mean (SD)	2.5 (1.4)	45.2 (23.4)	89.6 (3.0)	97.7 (1.5)	99.1 (1.3)	99.2 (1.3)	<0.001[a]	55.1 (30.4)
Median (IQR)	2.8 (1.8-3.9)	48.5 (28.1-68.9)	90.0 (87.5-92.5)	97.8 (96.5-99.1)	99.9 (98.4-101.3)	100 (98.5-101.5)		54.7 (25.9-83.4)
Socio-economic level								
N (%):								

High	16 (0.8)	259 (0.7)	28 (0.4)	18 (0.3)	5 (0.6)	0 (0.0)	0.053[b]	326 (0.6)
Middle	500 (26.2)	9,977 (25.5)	1,550 (24.6)	1,339 (25.9)	215 (25.6)	85 (26.2)		13,666 (25.4)
Low	1,144 (59.9)	24,387 (62.2)	3,987 (63.2)	3,234 (62.6)	529 (62.9)	204 (62.8)		33,485 (62.3)
missing	249 (13.0)	4,553 (11.6)	746 (11.8)	573 (11.1)	92 (10.9)	36 (11.1)		6,249 (11.6)
District of residency								
N (%):								
Central	51 (2.7)	632 (1.6)	119 (1.9)	87 (1.7)	20 (2.4)	10 (3.1)	<0.001[b]	919 (1.7)
Northern	402 (21.1)	8,482 (21.7)	1,201 (19.0)	934 (18.1)	146 (17.4)	46 (14.2)		11,211 (20.9)
Haifa	479 (25.1)	9,487 (24.2)	1,525 (24.2)	1,281 (24.8)	193 (22.9)	81 (24.9)		13,046 (24.3)
Sharon-Shomron	301 (15.8)	6,493 (16.6)	1,077 (17.1)	1,020 (19.8)	177 (21.0)	65 (20.0)		9,133 (17.0)
Dan-PT	50 (2.6)	1,238 (3.2)	212 (3.4)	180 (3.5)	15 (1.8)	11 (3.4)		1,706 (3.2)
Jerusalem	229 (12.0)	6,681 (17.1)	1,304 (20.7)	1,043 (20.2)	201 (23.9)	76 (23.4)		9,534 (17.7)
Southern	397 (20.8)	6,163 (15.7)	873 (18.8)	619 (12.0)	89 (10.6)	36 (11.1)		8,177 (15.2)

85th-94.9th percentile, obesity- BMI ≥95th percentile, not including class 2 and class 3 obesity, class 2 obesity- BMI ≥120% to <140% of the 95th percentile or BMI ≥35 to <40 kg/m^2, class 3 obesity- BMI ≥140% of the 95th percentile or BMI ≥40 kg/m^2. SD-standard deviation, IQR- interquartile range, [a]Kruskal-Wallis test, [b]Chi-square test.

Table S4. Risk estimates of the association between adolescent weight category and incident hypertension and diabetes mellitus type 2 in young adulthood

	Total	Weight categories in adolescence						P
		Underweight	Normal	Overweight	Obese	Class 2 obesity	Class 3 obesity	
Hypertension								
Participants in category, N	53,525	1,901	39,091	6,286	5,106	826	315	
Incident cases, N	848	13	376	151	231	49	28	
Follow-up, years, Mean (SD)	11.5 (1.7)	11.6 (1.5)	11.5 (1.6)	11.4 (1.8)	11.4 (2.1)	11.0 (2.3)	11.0 (2.3)	<0.001
Person-years of follow-up	613,409.7	22,129.44	448,951.2	71,348.3	58,400.1	9,111.2	3,469.4	
Incidence (95% CI) (per 10^{-5} person-years)	138.2 (129.1-147.9)	58.7 (31.3- 100.5)	83.8 (75.5 92.7)	211.6 (179.2-248.2)	395.5 (346.2-450.0)	537.8 (397.9-711.0)	807.1 (536.3-1166.4)	<0.001
Age at end of follow-up, years, Mean (SD)	29.7 (1.5)	29.8 (1.3)	29.7 (1.4)	29.6 (1.7)	29.5 (1.9)	29.4 (2.2)	29.4 (2.2)	<0.001
Age at diagnosis, years, Mean (SD)	26.0 (4.3)	26.2 (4.3)	26.7 (4.0)	25.9 (4.3)	25.4 (4.4)	24.2 (4.3)	24.5 (4.2)	<0.001
Adult BMI, kg/m^2, Mean (SD)	26.7 (6.1)	21.0 (4.0)	25.1 (4.7)	30.1 (4.9)	33.9 (5.7)	39.9 (6.1)	43.7 (8.3)	<0.001
HR (95% CI), P-value		0.70 (0.40-1.21) 0.201	Reference	2.53 (2.10-3.06) <0.001	4.70 (3.99-5.53) <0.001	6.47 (4.80-8.71) <0.001	9.71 (6.61-14.26) <0.001	

aHR (95% CI)<br>P-value		0.85 (0.48-1.51)<br>0.588		1.89 (1.56-2.30)<br><0.001	2.64 (2.20-3.17)<br><0.001	2.37 (1.70-3.31)<br><0.001	2.80 (1.82-4.30)<br><0.001	
Diabetes Mellitus type 2								
Participants in category, N	53,489	1,898	39,029	6,282	5,126	836	318	
Incident cases, N	839	9	415	139	200	46	30	
Follow-up, years, Mean (SD)	11.5 (1.6)	11.6 (1.5)	11.5 (1.6)	11.4 (1.8)	11.5 (1.9)	11.2 (1.9)	11.0 (2.3)	<0.001
Person-years of follow-up	613,790.8	22,106.8	448,303.8	71,487.8	59,009.1	9,371.1	3,512.2	
Incidence (95% CI) (per 10^{-5} person-years)	136.7 (127.6-146.3)	40.7 (18.6-77.3)	92.6 (83.9-101.9)	194.4 (163.5-229.6)	338.9 (293.6-389.3)	490.9 (359.4-654.8)	854.2 (576.3-1219.4)	<0.001
Age at end of follow-up, years, Mean (SD)	29.7 (1.5)	29.8 (1.3)	29.8 (1.4)	29.7 (1.6)	29.6 (1.7)	29.5 (1.6)	29.4 (2.1)	<0.001
Age at diagnosis, years, Mean (SD)	25.5 (3.3)	23.9 (3.0)	25.6 (3.2)	25.7 (3.3)	25.3 (3.3)	25.7 (3.4)	24.6 (3.5)	0.196
Adult BMI, kg/m^2, Mean (SD)	26.7 (6.1)	21.0 (4.0)	25.0 (4.7)	30.1 (4.9)	33.9 (6.5)	40.0 (6.1)	43.8 (8.3)	<0.001
HR (95% CI),<br>P-value		0.44 (0.23-0.85)<br>0.014	Reference	2.11 (1.74-2.55)<br><0.001	3.64 (3.08-4.31)<br><0.001	5.35 (3.95-7.26)<br><0.001	9.32 (6.43-13.50)<br><0.001	
aHR (95% CI),<br>P-value		0.65 (0.33-1.25)<br>0.198		1.46 (1.20-1.77)<br><0.001	1.82 (1.51-2.18)<br><0.001	1.63 (1.17-2.27)<br>0.004	1.97 (1.31-2.96)<br>0.001	

Underweight- BMI <5th percentile, normal weight- BMI 5th-84.9th percentile, overweight- BMI 85th-94.9th percentile, obese- BMI ≥95th percentile, not including class 2 and class 3 obesity, class 2 obesity- BMI ≥120% to <140% of the 95th percentile or BMI ≥35 to <40 kg/m^2, class 3 obesity- BMI ≥140% of the 95th percentile or BMI ≥40 kg/m^2. SD-standard deviation, 95% CI- 95% of the confidence interval, HR- Hazard ratio, aHR- Hazard ratio adjusted to sex, socio-economic level, district of residency and adult BMI

Table S5. Risk estimates of the association between adolescent weight category and incident ischemic stroke, myocardial infarction, and heart failure in young adulthood.

		Weight category in adolescence			
	Total	Nonobese	Overweight	Obese	P
Composite endpoint: ischemic stroke, myocardial infarction, and heart failure					
Participants in category, N	53,707	41,073	6,308	6,326	
Incident cases, N	169	114	32	23	
Follow-up, years, Mean (SD)	11.5 (1.5)	11.5 (1.5)	11.5 (1.6)	11.6 (1.6)	<0.001
Person-years of follow-up	619,325.5	473,452.4	72,240.1	73,633.0	
Incidence (95% CI) (per 10^{-5} person-years)	27.3 (23.3-31.7)	24.1 (19.9- 28.9)	44.3 (30.3- 62.5)	31.2 (19.8- 46.9)	0.007
Age at end of follow-up, years, Mean (SD)	29.8 (1.3)	29.8 (1.3)	29.7 (1.4)	29.8 (1.4)	0.043
Age at diagnosis, years, Mean (SD)	25.4 (3.2)	25.5 (3.1)	25.2 (3.1)	25.4 (3.8)	0.785
Adult BMI, kg/m^2, Mean (SD)	26.7 (6.2)	24.9 (4.8)	30.1 (4.9)	35.2 (6.6)	<0.001
HR (95% CI)			1.85 (1.25-2.73)	1.29 (0.82-2.02)	
P-value			0.002	0.269	
aHR[§](95% CI)		Reference	1.70 (1.12-2.57)	0.99 (0.58-1.69)	
P-value			0.012	0.962	
aHR[§§] (95% CI)			1.64 (1.08-2.50)	0.81 (0.46-1.42)	
P-value			0.020	0.459	
RR (95% CI)			1.79 (1.17-2.67)	1.35 (0.84-2.10)	
P-value			0.006	0.193	
aRR[§] (95% CI)			1.68 (1.07-2.58)	1.07(0.61-1.86)	
P-value			0.020	0.798	
aRR[§§] (95% CI)			1.64 (1.05-2.51)	0.79 (0.43-1.41)	
P-value			0.025	0.440	
Individual components of the composite endpoint: ischemic stroke					
Participants in the category	53,719	41,081	6,309	6,329	
Incident cases, N	68	47	16	5	
Follow-up, years, Mean (SD)	11.5 (1.5)	11.5 (1.5)	11.5 (1.6)	11.6 (1.5)	<0.001
Person-years of follow-up	619,909.0	473,840.2	72,345.3	73,723.5	

Incidence (95% CI) (per 10^{-5} person-years)	11.0 (8.5-13.9)	9.9 (7.3- 13.2)	22.1 (12.6- 35.9)	6.8 (2.2- 15.8)	0.007
Age at end of follow-up, years, Mean (SD)	29.8 (1.3)	29.8 (1.3)	29.8 (1.4)	29.8 (1.3)	0.107
Age at diagnosis, years, Mean (SD)	25.7 (3.0)	25.7 (2.9)	26.2 (2.5)	24.2 (5.1)	0.852
Individual components of the composite endpoint: myocardial infarction					
Participants in category	53,723	41,082	6,311	6,330	
Incident cases, N	35	23	6	6	
Follow-up, years, Mean (SD)	11.5 (1.5)	11.5 (1.5)	11.5 (1.6)	11.7 (1.5)	<0.001
Person-years of follow-up	620,081.0	473,941.1	72,386.5	73,753.3	
Incidence (95% CI) (per 10^{-5} person-years)	5.6 (3.9- 7.9)	4.9 (3.1- 7.3)	8.3 (3.0- 18.0)	8.1 (3.0- 17.7)	0.327
Age at end of follow-up, years, Mean (SD)	29.8 (1.3)	29.8 (1.3)	29.8 (1.4)	29.8 (1.3)	0.106
Age at diagnosis, years, Mean (SD)	25.6 (3.5)	25.3 (3.7)	24.4 (3.6)	27.9 (1.5)	0.175
Individual components of the composite endpoint: heart failure					
Participants in the category	53,717	41,080	6,310	6,327	
Incident cases, N	76	50	14	12	
Follow-up, years, Mean (SD)	11.5 (1.5)	11.5 (1.5)	11.5 (1.6)	11.6 (1.5)	<0.001
Person-years of follow-up	619,909.0	473,810.5	72,342.1	73,684.2	
Incidence (95% CI) (per 10^{-5} person-years)	12.3 (9.7- 15.3)	10.6 (7.8- 13.9)	19.3 (10.6-32.5)	16.3 (8.4- 28.4)	0.079
Age at end of follow-up, years, Mean (SD)	29.8 (1.3)	29.8 (1.3)	29.8 (1.4)	29.8 (1.3)	0.080
Age at diagnosis, years, Mean (SD)	25.3 (3.1)	25.5 (2.9)	24.4 (3.3)	25.4 (3.7)	0.569

Underweight- BMI <5th percentile, normal weight- BMI 5th-84.9th percentile, overweight- BMI 85th-94.9th percentile, obese- BMI ≥95th percentile, not including class 2 and class 3 obesity, class 2 obesity- BMI ≥120% to <140% of the 95th percentile or BMI ≥35 to <40 kg/m^2, class 3 obesity- BMI ≥140% of the 95th percentile or BMI ≥40 kg/m^2. SD-standard deviation, 95% CI- 95% of the confidence interval, HR- Hazard ratio, aHR- Hazard ratio adjusted to sex, socio-economic level, district of residency and adult BMI.

Figure S1: Flowchart of the study cohort selection.

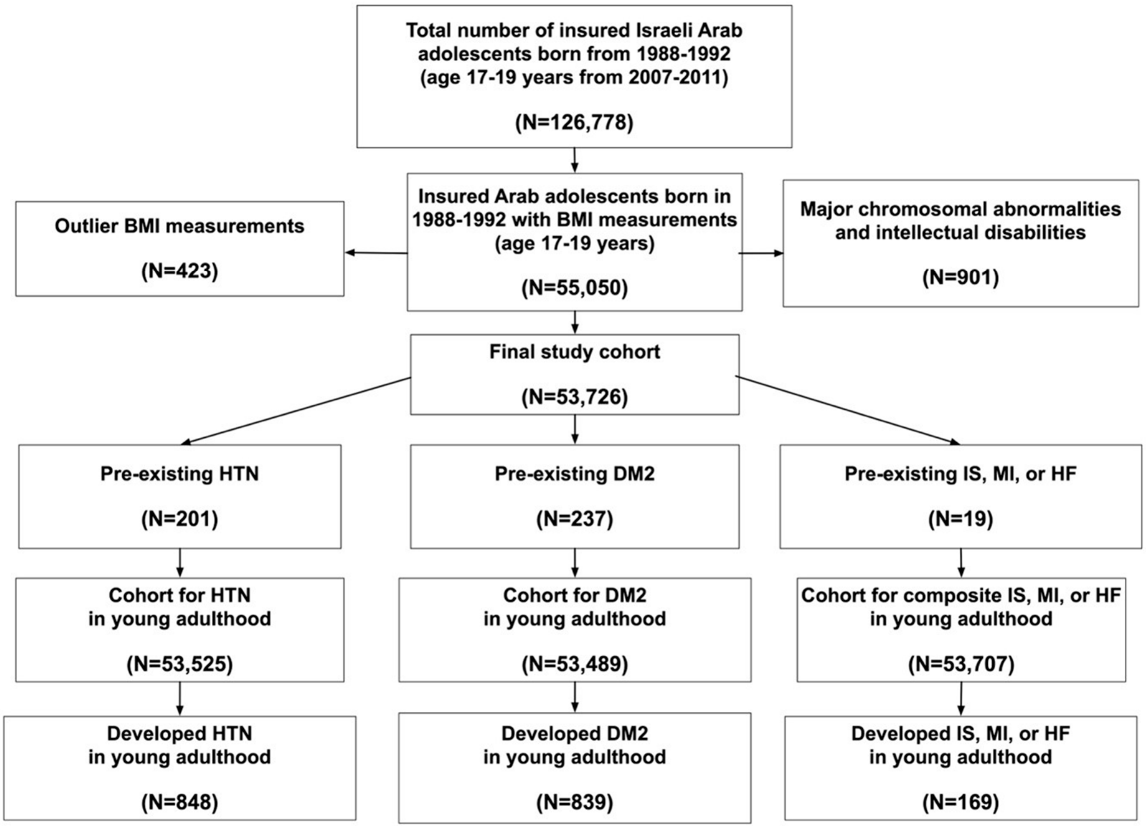

Supplement: Supplementary file 1 [file jcm-13-05382-s001.zip › jcm-3173435-supplementary.pdf]
